# Parkinson’s Is Time on Your Side? Evidence for Difficulties with Sensorimotor Synchronization

**DOI:** 10.3389/fneur.2015.00249

**Published:** 2015-11-27

**Authors:** Marta M. N. Bieńkiewicz, Cathy M. Craig

**Affiliations:** ^1^ISM, Faculté des Sciences du Sport, Université d’Aix-Marseille, Marseille, France; ^2^School of Psychology, Queen’s University Belfast, Belfast, UK

**Keywords:** basal ganglia, temporal control, sensorimotor synchronization, Fitts’ law, event-based timing, index of difficulty, PD, motor sychronisation

## Abstract

There is lack of consistent evidence as to how well PD patients are able to accurately time their movements across space with an external acoustic signal. For years, research based on the finger-tapping paradigm, the most popular paradigm for exploring the brain’s ability to time movement, has provided strong evidence that patients are not able to accurately reproduce an isochronous interval [i.e., Ref. (1)]. This was undermined by Spencer and Ivry (2) who suggested a specific deficit in temporal control linked to emergent, rhythmical movement not event-based actions, which primarily involve the cerebellum. In this study, we investigated motor timing of seven idiopathic PD participants in event-based sensorimotor synchronization task. Participants were asked to move their finger horizontally between two predefined target zones to synchronize with the occurrence of two sound events at two time intervals (1.5 and 2.5 s). The width of the targets and the distance between them were manipulated to investigate impact of accuracy demands and movement amplitude on timing performance. The results showed that participants with PD demonstrated specific difficulties when trying to accurately synchronize their movements to a beat. The extent to which their ability to synchronize movement was compromised was found to be related to the severity of PD, but independent of the spatial constraints of the task.

## Introduction

Parkinson’s disease (PD) is a neurodegenerative disease of the substantia nigra pathways in the brain that affects 4.5 million Europeans, a number that is estimated to double by 2030 (3). As decreased dopamine levels debilitate normal motor function, people with PD tend to move 30–40% slower (bradykinesia) than healthy adults with a movement range that is often underscaled (4). The underscaled (hypometria) movement is characterized by decreased amplitude when compared to the movement of a neurologically healthy adult (i.e., shorter stride, smaller handwriting). This is also accompanied by an irregular pattern of force unfolding over time, with patients needing twice as much time to achieve peak force compared to healthy adults. All of these symptoms associated with the disease combine together to cause particular difficulties with performing everyday actions such as walking (characterized by a shuffling gait and difficulty with turning), dressing, handwriting (5), or using a computer mouse (6).

An important question is how well patients can adjust their movements to meet the spatio-temporal demands of a given task, in particular being at the right place at the right time. In the early 1960s, Draper and Johns (7) reported that people with PD have the same velocity for their movements regardless of movement amplitude. This was later questioned in a study by Teasdale et al. (8) and also by Mazzoni et al. (9) who both showed that although patients move slower, they can modify their movement times (MT) and maintain similar levels of accuracy to that observed in healthy adults. Furthermore, Sanes (10) noted that patients with PD struggle with high velocity movements over longer distances; with a notable breakdown in the ability to perform the task when the accuracy demands increase being observed. In a similar vein, Rand (11) reported particular difficulties in PD participants with the temporal aspects of motor control (the deceleration phase and higher movement variability) when a spatial accuracy constraint was introduced in a pointing task.

In general, healthy adults tend to move faster in order to intercept the target when the distance between the hand and object is greater (12). The velocity of every aiming movement is partially defined by Fitts’ law that describes a movement based speed accuracy trade-off (13, 14). Interestingly patients do not show differences in the time they need to initiate movement when compared to healthy adults in reaction time aiming tasks (15). However, in reciprocal aiming longer MT and dwelling time were found for patients in late stage of PD compared to controls (16). Importantly, none of these studies considered the spatial and informational constraints of the task in the context of the temporal accuracy of the movement, something that is very important in sensorimotor synchronization.

Controlling a movement in many instances also requires that it is controlled with respect to external events in the future. A plethora of research suggests that dopamine plays an important part in temporal processing and prediction [see Ref. (17) for review]. Studies on rats demonstrate that lesions of the hippocampus result in increased dopamine release to the striatum, which disrupts timing in both second and minute scales (18). In humans, administration of a dopamine agonist (haloperidol) to healthy adults affects our ability to accurately discriminate temporal durations under 500 ms (19).

For decades, research based on the finger-tapping paradigm has provided strong evidence that patients are not able to accurately reproduce an isochronous interval (1, 20). Spencer and Ivry (2) have shed new light on those results in their study that uses both finger-tapping and circle-drawing. In both tasks, when patients executed movements in an intermittent, discontinuous manner (with a pause after each motor response), they performed as well as healthy adults. However, if patients with PD performed the same circle-drawing movement in a continuous manner, they exhibited increased temporal variability (2). This suggests that the timing of movements, which emerge and require intrinsic temporal control may be controlled differently from the timing of movements that are linked to a sensory event, such as coupling a movement to the sounding of a beat. These findings gave rise to the idea that event-based timing (moving to a beat) operates independently from basal ganglia structures and relies on preserved cerebellar functions instead (21). However, a report by Diedrichsen et al. (22) suggests that PD patients have difficulty with accurate synchronization, as a result of problems with error correction processes. In a similar vein, Grahn and Brett (23) reported impaired ability of PD patients to discriminate complex rhythmical structures. Other studies measuring perception of time intervals without using a motor response, i.e., where patients verbalize whether two time intervals are different or not, failed to find evidence for differences between people with PD and healthy adults (24). This contradictory evidence leaves many questions unanswered about the temporal control of the movement in PD.

In this study, we aimed to investigate whether people with PD are able to synchronize an aiming movement toward a target to the sounding of an external acoustic beat. We wanted to see how the spatial-temporal control of the movement is influenced by both the spatial demands of the task (e.g., cover the distance between two target zones) and the informational load of the task [i.e., the index of difficulty (ID) for accurate interception], as characterized by Fitts’ law. Previous research also suggests that a 2.5-s inter-beat interval is the upper threshold for accurate timekeeping, with longer durations causing a breakdown in temporal control (25). To investigate this further with PD participants, we deliberately used longer inter-beat intervals (1.5 and 2.5 s) than the standard metronome frequencies used in finger-tapping studies [usually under 1 s; (1, 20, 22)]. Performance of PD patients was compared to the performance of a group of healthy adults (26). This group was deemed appropriate as a study conducted by Drewing (27) demonstrated no significant difference in synchronization ability (error correction of phase relation) across the life span. Furthermore, the temporal accuracy in synchronization was found to be stable from adolescence (approximately age 15) into old adulthood (59–88 years). In addition, Elliott et al. (28) demonstrated that elderly adults (63–80 years) matched young controls in their synchronization performance when moving in time with an isochronous metronome.

## Materials and Methods

### Participants and Medical Assessment

Seven right-handed, idiopathic PD participants (one female and six males; average age *M* = 63.4, SD = 5.9) volunteered to participate in the experiment. All of the participants were right-hand dominant and had normal or corrected to normal vision. Participants were recruited through the out-patient clinic at Belfast City Hospital. Ethical approval was granted by the Office for Research Ethics Committees Northern Ireland.

All participants were tested in the morning or early afternoon depending on their optimal functioning and mobility levels during the day. There was no change in their medication schedule to avoid any hazards in a non-medical setting (Psychology Laboratory, Queen’s University of Belfast). Before the experiment began, the medical condition of each patient was assessed by a qualified PD nurse. The assessment comprised of: the Hoehn and Yahr scale (29), the Unified PD Rating Scale (30), an Objective Dyskinesia Rating Scale (31), and the Mini-Mental State Examination (MMSE) (32). The Hoehn and Yahr scale (H&Y) rating scale classifies severity of the disease starting from 0 (no signs of the disease), 1 – unilateral signs, 2 – mild stage with bilateral signs, 3 – moderate symptoms with postural instability, 4 – severe disability, ability to walk preserved, up to 5 – the most advanced stage, where patients are unable to move without assistance and are usually using a wheelchair (29). The Unified PD Rating Scale (UPDRS) is an additional scale, applied to measure disability and severity of the symptoms in PD patients, in both clinical and research settings (30). It consists of four parts, examining various spheres of functioning and well-being (Cognition, Mood, and Behavior; Activities of Daily Living; Motor Examination; Complication of disease and Therapies). In this study, patients were examined using all parts of the UPDRS. The higher the score in UPDRS, the more advanced the symptoms of the disease. In addition, the state of all patients was assessed by the nurse as either being “On or Off.” “On” describes a phase of the day when the symptoms of the disease are partially alleviated by the dopaminergic treatment, as opposed to “Off” where they experience a “wearing off” of the medication’s effect. All patients were assessed as being “On” before testing. Severity of dyskinesias was evaluated using the Objective Dyskinesia Rating Scale (31). Patients were asked to perform three motor tasks: lift a cup, put a coat on and walk. Their performance was assessed on a 5-point scale (0 – dyskinesia absent during the motor task, to 5 – violent dyskinesia debilitating motor task performance) and summed for all three tasks. Additionally, the MMSE was used to screen for cognitive impairment and confirm patients’ ability to understand the task and consent to the study. The MMSE is a widely used scale measuring orientation to time and place, working and short-term memory and language (32). A score of over 25 points (with maximum of 30) is interpreted as normal (preserved cognitive functioning). Lower scores suggest increased probability of cognitive impairment or dementia of a mild level (21–24 points), moderate level (10–20 points), or severe level (below nine points) (33). Clinical features of patients are presented in Table 1.

**Table 1:** **Overview of the medical assessment of participants**.

Participant’s code	Hoehn and Yahr rating	UPDRS (ON)	Age	Sex	Years from diagnosis	Goetz dyskinesia scale	MMSE
PD1	1.5	20	70	M	1	0/12	29/30
PD2	1.5–2	29	66	M	4	0/12	30/30
PD3	1.5–2	48	66	M	5	0/12	30/30
PD4	3–4	58	58	F	16	5/12	29/30
PD5	3–4	68	53	M	12	5/12	29/30
PD6	4	79	64	M	9	7/12	28/30
PD7	4	89	67	M	14	0/12	24/30

*M, male; F, female; UPDRS, Unified PD Rating Scale; MMSE, The Mini-Mental State*.

Participants were of a varied medical state according to the PD motor disability scales (H&Y, UPDRS) and years of the disease. The participant coded as PD7 was at the most advanced stage of the disease (H&Y stage 4, UPDRS 89) and was the only patient who had a MMSE score 1 point below the norm (lower range of mild cognitive impairment). In this case written consent was taken from the spouse of the patient.

The control group consisted of eleven right-handed, healthy adults (4 females and 7 males; average age *M* = 24.8 years, SD = 2.5 years), who volunteered to participate in the experiment [same participants as in Ref. (26)]. All participants were right-hand dominant with normal or corrected to normal vision and no neurological history.

### Apparatus

**Figure 1:**
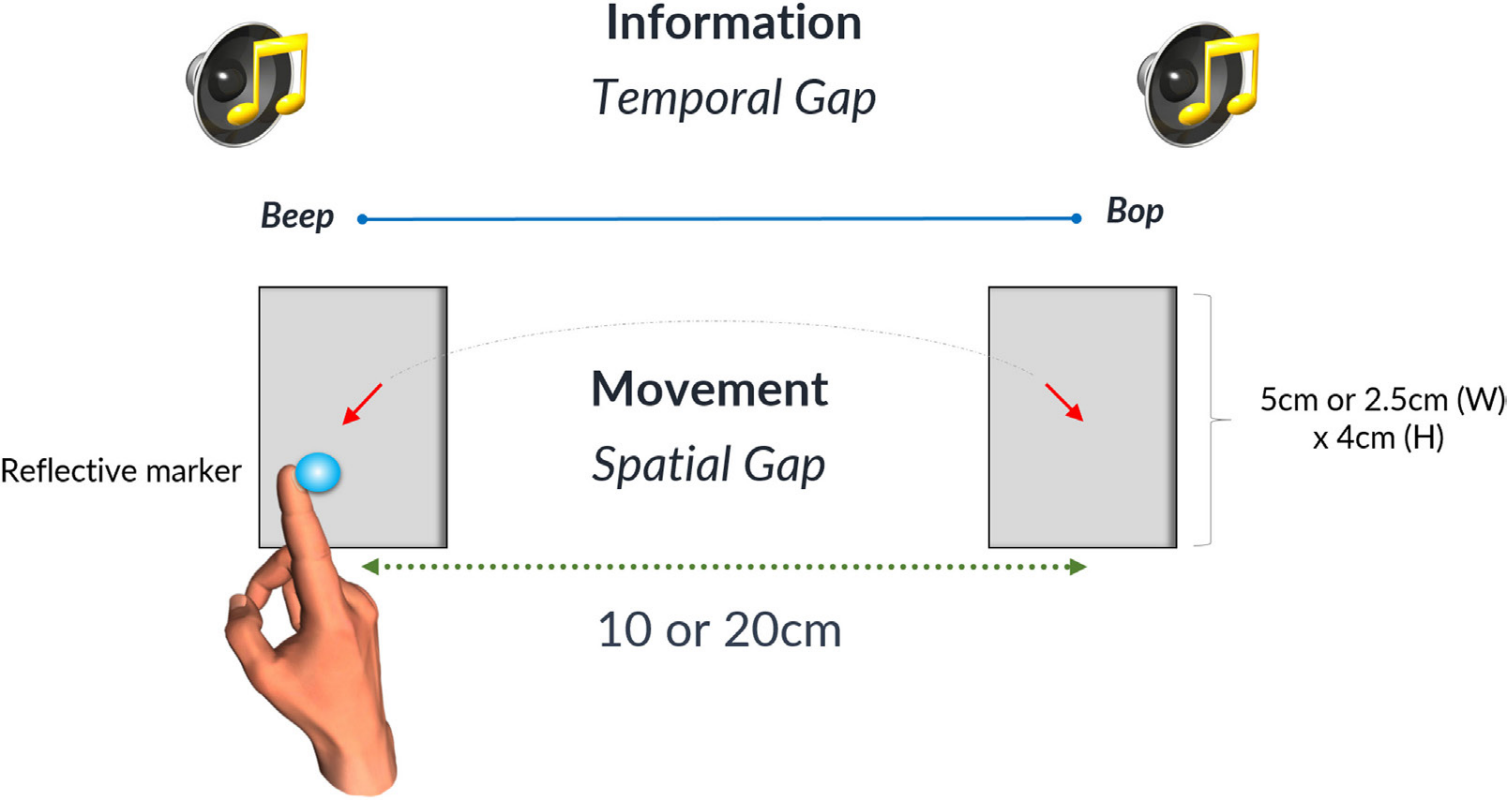
**The gray rectangles denote the two target zones that were either 5 or 2.5 cm wide**. Likewise the spatial gap between targets could be either 10 or 20 cm. The time interval between the occurrences of the successive beats was defined as a temporal gap on the diagram. Reproduced from Ref. (26). A diagram illustrating the experimental setup. With kind permission from Springer Science+Business Media: Bieńkiewicz MMN, Rodger MWM, Craig CM. Timekeeping strategies operate independently from spatial and accuracy demands in beat-interception movements. Exp Brain Res (2012) 222:241–53. Figure 1.

The setup was identical to that outlined in the study of Bieńkiewicz et al. (26) with the only difference being two inter-beat durations used (1.5 and 2.5 s) instead of three. Inter-beat intervals were displayed using two 50 ms sine tones: “beep” (500 Hz) and “bop” (700 Hz) synthesized using Adobe Audition and timed using a MP3 player (providing 1 ms precision of replays). Signals were presented through noise-isolated headphones at fixed volume levels. To explore the effects of spatial accuracy on the temporal control of movement, four sets of spatial targets were presented on laminated A3 printout displays. The width and distance between targets varied across sets to control for the ID, represented by Fitt’s law as:

(1) $$\text{ID}={\text{log}}_{\text{2}}\left(\text{2D}/\text{W}\right)$$

where W is the width of the target and D is the distance between targets for one interceptive movement (13). Spatial conditions are summarized in the table below (Table 2). Participants wore a thimble on their index finger that had a reflective marker placed at the end. The thimble minimized the friction between the finger and the laminated targets and ensured that the finger could move easily back and forth between targets (see Figure 1). Motion data were recorded using 8 Oqus 300 cameras (Qualisys Motion Capture System) sampling at a frequency of 200 Hz with a spatial accuracy close to ±0.1 mm. Before the start of each experimental condition coordinates of the target zones were recorded to calibrate the motion capture data with respect to the target position. An analog input from the MP3 player was recorded through a Qualisys Analog Board to allow for the temporal alignment of the timing of the sound events and the movement data between targets.

**Table 2:** **An overview of the target sizes and widths and corresponding IDs used in the experiment**.

Condition	Distance between the targets (cm)	Size of the targets (W **×** H)	Index of difficulty (ID)
I	10	2.5 cm × 4 cm	3
II	10	5 cm × 4 cm	2
III	20	5 cm × 4 cm	3
IV	20	2.5 cm × 4 cm	4

*Note how for the same amplitude of movement (distance between the targets) conditions differed in terms of ID as a result of the different target sizes. However, conditions I and III had the same ID but different distances between the targets [reproduced from Ref. (26)]. With kind permission from Springer Science+Business Media: Bieńkiewicz MMN, Rodger MWM, Craig CM. Timekeeping strategies operate independently from spatial and accuracy demands in beat-interception movements. Exp Brain Res (2012) 222:241–53. Table 1*.

### Procedure

Participants were seated comfortably at a desk and were asked to move their index finger back and forth between the two targets displayed in front of them in such a way that the arrival of the finger in the target zone coincided with the sounding of the beat. A total of 10 beep interceptions in the target on the left side and 10 bop interceptions in the target on the right side were recorded per condition. The recording began on the eleventh pointing movement and stopped after a further 20 movements. A relatively low number of trials was selected to avoid fatigue in patients. Participants were not instructed on how fast they should move between the target zones. The order of the spatial conditions was randomized using the Latin squares method.

### Data Analysis

Data analysis and processing was consistent with our previous study (26). Selected aspects of temporal control were analyzed and included temporal variability (success rates, asynchrony, spread of error and central timekeeping variance), movement organization: including movement strategy, time and velocity. These measures allowed us to investigate in detail the spatio-temporal aspects of task performance and compare behavior between patients and controls used in a previous study. Positional data were filtered in MATLAB (34) using a second-order low-pass Butterworth filter at a frequency of 8 Hz from which the subsequent first derivative was taken for the velocity profile. Time stamps demarcating the end of the finger movement were computed as the first frame in which the velocity fell below 5% of peak velocity for each interceptive movement and where the finger was located within the boundaries of the target zone. We classified a trial as accurate if a participant stopped in the target zone within the temporal window of the sound event (50 ± 10 ms error). The spread of error measure was calculated as the absolute difference between the temporal errors (with relation to beat onset) made in consecutive trials. A detailed description of this measure is included in a previous study (26). For statistical analysis, mean values for each variable were calculated for each trial/participant and then analyzed using (2 × 4) Repeated Measures ANOVA, followed by *post hoc* comparisons.

## Results

### Success Rates

**Figure 2:**
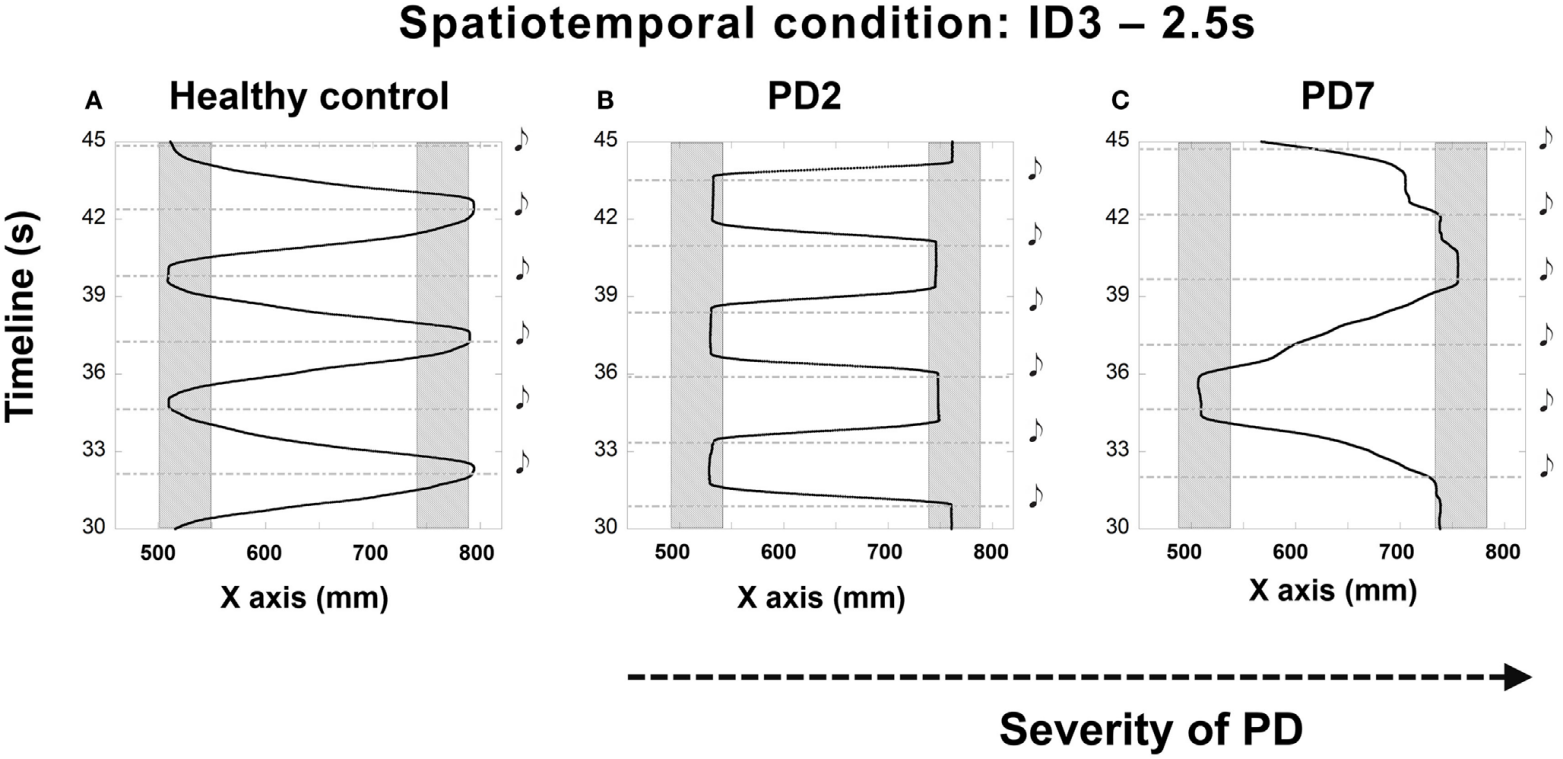
**Illustrations of the irregular synchronization patterns observed in patients at different stages of the disease compared to performance (A) of a healthy adult – PD2 [H&Y 1.5–2 (B)] and PD7 [H&Y 4 (C)]**. **(B)** shows the trajectory of the movement of patient PD2 who treated the sound as a cue for starting to move instead of a signal to stop. Patient PD7, at a more advanced stage of the disease **(C)** demonstrated a striking inability to synchronize the movement to the beats. Depicted trials were for the 2.5 s condition and ID3, 20 cm distance. Shaded rectangles denote target zones. Black musical denotes demarcate the onset of the beat.

The majority of participants found it challenging to synchronize their movement to the sounding of the beats. Those difficulties were expressed through early or late arrival in the target zones. In some trials, participants demonstrated a consistent pattern of initiating movement at beat onset instead of attempting to control their movements prospectively such that they would stop in the target zone at the same time as beat onset (see Figures 2A,B). Participant PD7 (with the most advanced PD symptoms in the tested sample) had to stop after just two trials as he reported that he could not get “into the swing” of the experiment and could not anticipate beat onset (see Figure 2C).

As the distance between the two target zones determined movement amplitude, temporal accuracy could be explored by manipulating the spatial requirements of the movement needed to complete the task. We found that PD patients were significantly less successful than the controls at synchronizing their movements to the onset of the beats [Repeated Measures ANOVA Between-Subject Effect *F*(1,13) = 55.002, *p* < 0.001, η^2^ = 0.786] (see Table 3 and Bieńkiewicz et al. (26)], but kept their movement spatially accurate by reaching target zones.

**Table 3:** **Percentage of high accuracy trials within the temporal window of 70 ms (50 ± 10 ms)**.

Interval duration								
**Participant’s code**	**1.5 s**				**2.5 s**			
Index of difficulty	ID 2	ID 3	ID 3	ID 4	ID 2	ID 3	ID 3	ID 4
Distance between targets (cm)	10	10	20	20	10	10	20	20

PD1 (%)	20	0	5	5	0	0	0	0
PD2 (%)	0	0	0	0	0	0	0	0
PD3 (%)	5	5	0	5	5	5	9	6
PD4 (%)	5	5	11	13	5	10	0	0
PD5 (%)	14	0	5	5	0	5	7	5
PD6 (%)	18	11	5	20	0	8	5	6
PD7 (%)	0	–	–	–	0	–	–	–
PD participants (%)	9	4	4	8	1	5	4	3
Healthy controls* (%)	12	11	9	7	12	11	12	17

A *s*ummary of the percentages of high accuracy trials for each participant compared to the average healthy adult performance (*n* = 11; bottom line). Participants are listed according to their UPDRS rating (from the lowest score – to the highest). Participants PD7 was not able to proceed with the rest of the trials. Note how with longer interval duration – 2.5 s, patients had lower success rates than the healthy adults* [sample from Ref. (26)].

Problems with synchronization, revealed by poor success rates in temporal accuracy, were further investigated by analyzing synchronization errors. Participants struggling to synchronize with the beat could either arrive in the target zone too early, underestimating the duration of the inter-beat interval, or too late, overestimating the duration of the inter-beat interval. Therefore, if they arrived and stopped in the target zone more than 10 ms before the sound event (less than −10 ms), the trial was classified as negatively asynchronous. If they arrived and stopped in the target zone 10 ms after the occurrence of the beat (i.e., >60 ms – duration of the sound 50 + 10 ms error) the trial was classified as positively asynchronous. The cut-off for temporal accuracy was set arbitrarily based on previous literature (25).

### Negative Asynchrony

Arriving and stopping in the target zone before the sound event implies that the participant’s could not accurately anticipate when the sound event was going to happen. Our tested sample of PD patients had 40% more negative asynchronies than a group of healthy controls Bieńkiewicz et al. (26) (see Table 4).

**Table 4:** **A summary table of negative asynchronies made by patients**.

Interval duration								
**Participant’s code**	**1.5 s**				**2.5 s**			
Index of difficulty	ID 2	ID 3	ID 3	ID 4	ID 2	ID 3	ID 3	ID 4
Distance between targets (cm)	10	10	20	20	10	10	20	20

PD1 (%)	70	75	52	90	100	100	100	100
PD2 (%)	100	100	95	100	95	100	100	100
PD3 (%)	10	14	6	5	19	20	45	33
PD4 (%)	14	29	53	70	76	71	67	71
PD5 (%)	10	48	67	11	0	0	7	38
PD6 (%)	50	33	14	0	67	23	62	56
PD7 (%)	38	_	_	_	18	_	_	_
PD participants (%)	42	50	48	46	54	52	64	66
Healthy controls* (%)	7	4	7	7	19	15	21	22

Percentages were calculated as the percentage of trials classified as negatively asynchronous with the inter-beat interval in each condition. In comparison to healthy adults* (*n* = 11) (26) patients had a higher ratio of negative asynchronies. Participants are listed according to their UPDRS rating (from the lowest score – to the highest).

The difference between patients and healthy controls was statistically significant [between subjects effects repeated measures ANOVA of ratio of negative asynchronies on two time intervals – *F*(1,15) = 14,434, *p* = 0.002, η^2^ = 0.490 – patients (time interval 1.5 s, *M* = 0.47, SD = 0.33, 2.5 s, *M* = 0.58, SD = 0.38) vs. healthy controls (time interval 1.5 s, *M* = 0.06, SD = 0.04, 2.5 s, *M* = 0.20, SD = 0.12)]. However, the differences between the 1.5 and 2.5 durations in the patient group were not statistically significant, *p* > 0.05.

In the patient group, we observed a significantly higher frequency of negative asynchrony errors over 300 ms before the occurrence of the beat in both interval durations than in healthy controls [Repeated Measures ANOVA Between Subjects Effects – patients vs. healthy controls on the ratio of negative asynchronies over −300 ms for two interval durations – *F*(1,14) = 5.498, *p* = 0.034, η^2^ = 0.282)]. An increase in the magnitude of negative asynchronies for longer interval durations supports our hypothesis that temporal control is compromised for longer interval durations.

### Positive Asynchrony

Arriving and stopping in the target zone after the sound event suggests that the inter-beat interval representation was too long and compromised the temporal control of the movement. Patients made fewer positive asynchronies than what was found with healthy controls in our previous study – [Repeated Measures ANOVA Between Subjects Effects *F*(1,15) = 21.43 *p* < 0.001, η^2^ = 0.588 – patients (time interval 1.5 s, *M* = 0.47, SD = 0.31, 2.5 s, *M* = 0.23, SD = 0.28) vs. healthy controls (time interval 1.5 s, *M* = 0.84, SD = 0.08, 2.5 s, *M* = 0.67, SD = 0.14) on the ratio of positive asynchronies per condition]. Nonetheless, at the more advanced stages of the disease we observed a tendency toward an increase in the ratio of positive asynchronies (see Table 5).

**Table 5:** **Summary table of positive asynchronies made by patients**.

Interval duration								
**Participant’s code**	**1.5 s**				**2.5 s**			
Index of difficulty	ID 2	ID 3	ID 3	ID 4	ID 2	ID 3	ID 3	ID 4
Distance between targets (cm)	10	10	20	20	10	10	20	20

PD1 (%)	10	25	43	5	0	0	0	0
PD2 (%)	0	0	5	0	5	0	0	0
PD3 (%)	85	81	94	90	76	75	45	61
PD4 (%)	81	67	37	17	19	19	33	29
PD5 (%)	76	52	29	84	100	95	87	57
PD6 (%)	32	56	82	80	33	69	33	39
PD7 (%)	62	–	–	–	82	–	–	–
PD participants (%)	49	47	48	46	45	43	33	31
Healthy controls* (%)	81	85	85	86	69	74	67	61

Percentages were calculated as the percentages of trials classified as an overestimation of inter-beat intervals in each condition. In comparison to healthy adults* [bottom row – sample taken from Ref. (26)] patients had a lower ratio of positive asynchronies. Participants are listed according to their UPDRS rating (from the lowest score – to the highest). Note how patients with more advanced stage PD have higher ratios of positive asynchronies.

The profiling of positive asynchronies revealed significantly higher values of error in the patient group – [Repeated Measures ANOVA Between Subjects Effects *F*(1,15) = 5.53 *p* = 0.03, η^2^ = 0.269 – patients (time interval 1.5 s, *M* = 0.38, SD = 0.37, 2.5 s, *M* = 0.51, SD = 0.31 vs. healthy controls (time interval 1.5 s, *M* = 0.11, SD = 0.09, 2.5 s, *M* = 0.27, SD = 0.15) on the ratio of positive asynchronies over 350 ms per temporal condition]. In other words, if patients overestimated the duration of the interval the magnitude of their error (in ms) was significantly greater than that of healthy adults. There was no difference in the patient group between the 1.5 and the 2.5 s interval durations and the ratio of positive asynchronies over 350 ms after the occurrence of the beat, *p* > 0.05.

### Temporal Variability

**Figure 3:**
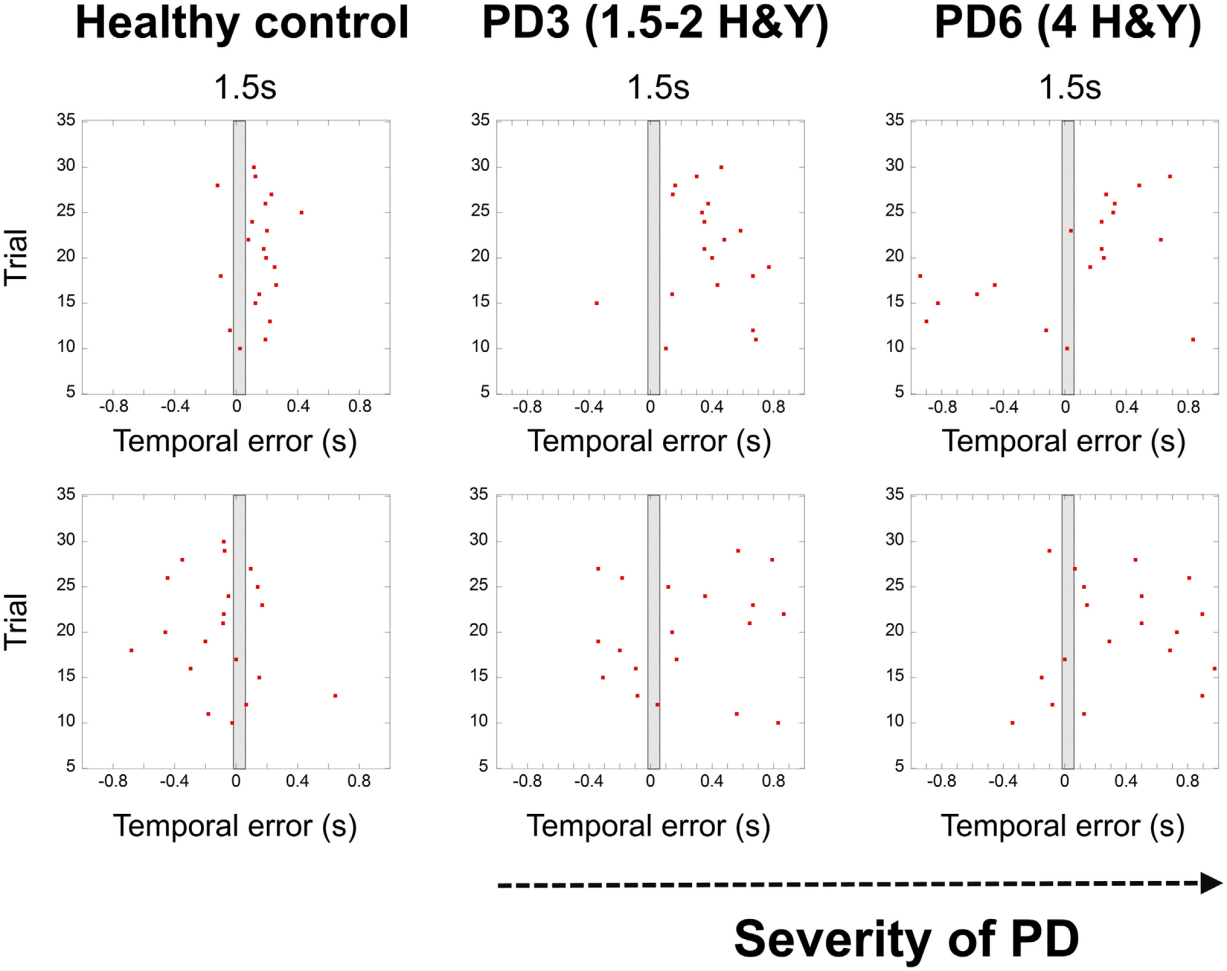
**Graphs showing the breakdown of temporal control with the severity of PD (HY – healthy control, patient – PD3 at stage 1.5–2 Hoehn and Yahr, patient PD6 at stage 4 Hoehn and Yahr)**. Red dots denote synchronization errors relative to the occurrence of a beat for each trial. The shaded rectangle in the middle represents the duration of the inter-beat interval. Red dots in the gray rectangle indicate high accuracy trials (within a temporal window of 70 ms). Depicted trials were for the 1.5 s and 2.5 s conditions and ID3, 20 cm distance.

**Figure 4:**
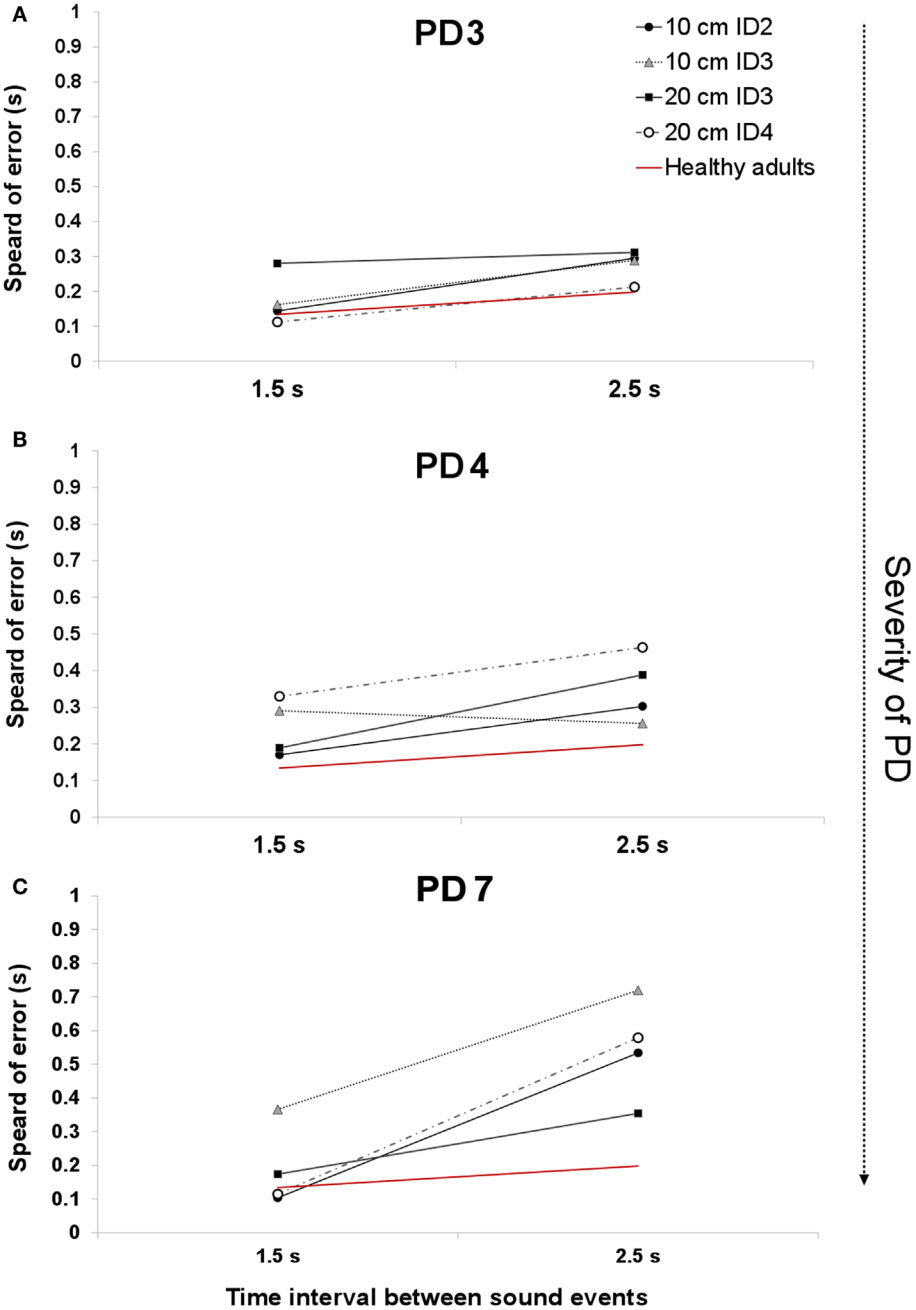
**An example of the average spread of error across two interval durations for patients PD3 [H&Y 1.5–2 (A)], PD4 [H& Y 3.5–4 (B)], and PD6 [H&Y 4 (C)]**. The red line denotes the average spread of error for healthy adults.

In Ref. (26), we demonstrated that the spread of error is a novel and robust measure of temporal variability for this task. In this study, we expected to find a larger spread of error for PD patients, showing a breakdown in their temporal control when compared to that of healthy adults. In healthy adults (26), we observed a pattern of increased temporal variability when synchronizing with longer interval durations compared to shorter 1.5 s durations. PD patients had significantly higher values in the spread of error than healthy adults [Repeated Measures ANOVA *F*(1,15) = 4.743, *p* = 0.003, η^2^ = 0.444]. We found a trend toward a higher spread of error values with more advanced stages of the disease (Figures 3 and 4).

The UPDRS total score strongly correlated with the measure of spread of error for the 1.5 s interval Spearman’s one-tailed ρ = 0.85, *p* = 0.007, and with the 2.5 s interval (ρ = 0.67, *p* = 0.05). Figure 4A illustrates the spread of error for a patient at a mild stage of the disease. For almost all of the display, the spread of error is higher than the average found for healthy controls, oscillating between 100 and 300 ms. With the severity of the disease (See Figure 4B) we observe an increase in the spread of error from 150 to 450 ms, where the increased variability in synchronization errors means greater difficulty with task performance. Interestingly, no effect of the informational load and/or the amplitude of movement was found in the spread of error, *p* > 0.05 (See Figure 4C). Although we expected that for more challenging spatial conditions (i.e., higher IDs), we would observe a larger spread of error, this was not found to be the case. The increase in the spread of error in PD was attributed to the temporal demands of the task.

### Movement Strategies

Preliminary analyses of MT revealed two different strategies of timekeeping that were consistent with our findings from the study on healthy controls. Our assumption that patients might reveal different movement strategies compared to those of healthy adults was not upheld. Three patients adopted a MT strategy and adjusted MT to the interval duration, while the other three varied their Waiting Time (WT strategy) in the target zones according to the interval duration. Patients in both groups were at varying stages of the disease. We identified MT in PD3, PD5, and PD6; and WT in PD1, PD2, and PD4. We were unable to identify a pattern of movement strategy in PD 7 – our most advanced patient.

**Figure 5:**
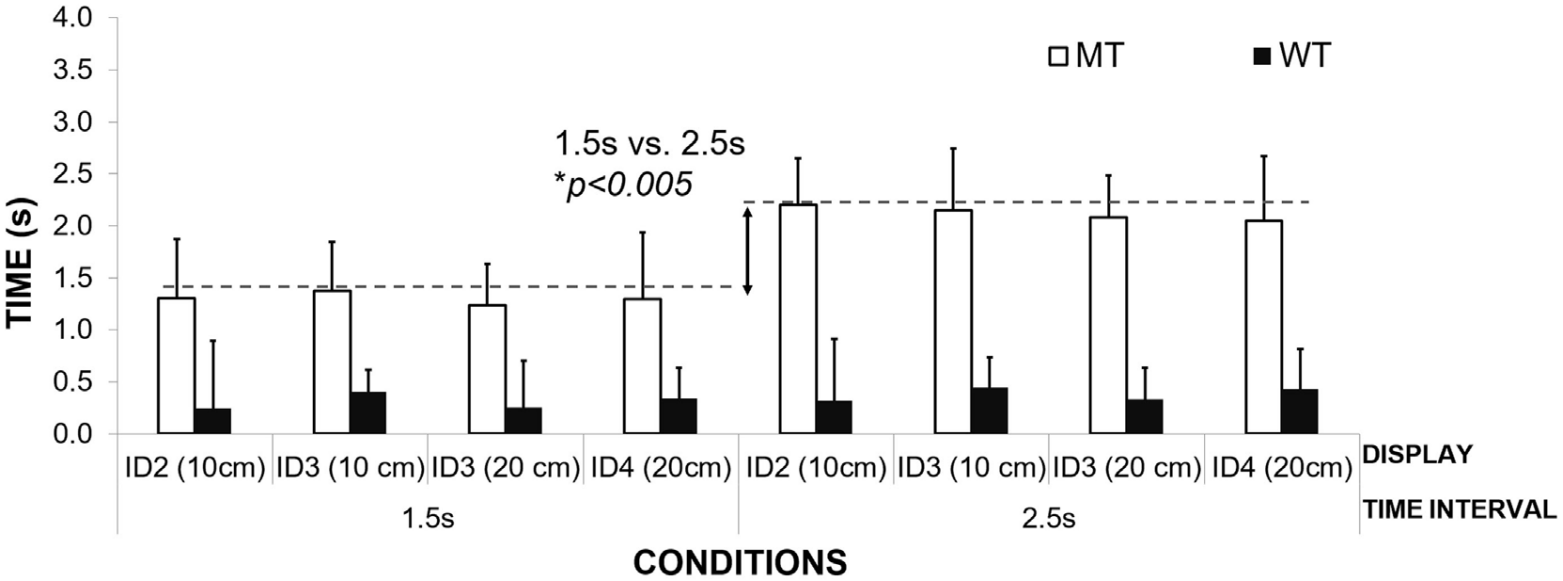
**A graph illustrating the mean movement times for participants in the MT strategy group**.

Figure 5 illustrates the differences in mean MT for the different interval durations in the MT group. Adjusting MT to the duration of the interval suggests that, similar to healthy adults, patients filled the inter-beat interval with movement. The MT was adjusted to the interval duration, and not the required movement amplitudes or the informational loads of the task (different IDs). MT was found to be significantly different for the two interval durations – 1.5 s (*M* = 1.30 s; SD = 0.05) and 2.5 s (*M* = 2.20 s, SD = 0.11) [repeated Measures ANOVA Effect on Interval Duration on MT *F*(1,2) = 218.95, *p* = 0.005, η^2^ = 0.99]. Patients did not show any differences in the mean MT when compared to healthy controls who used the same strategy (*M* = 1.23 s, SD = 0.02; *M* = 2.12 s, SD = 0.07 respectively to the interval duration) [between subjects effects repeated measures ANOVA – group healthy controls vs. patients *p* > 0.05]. No effect of IDs or movement amplitude was observed on the mean MT, *p* > 0.05.

**Figure 6:**
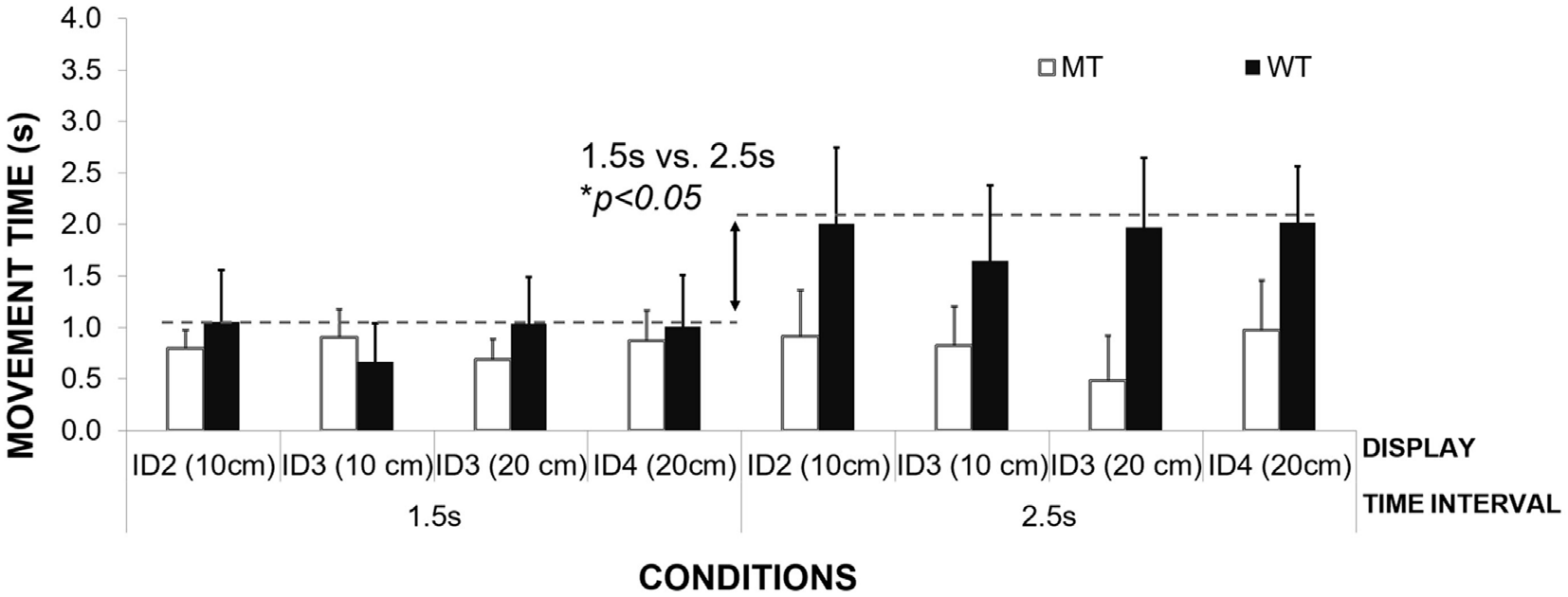
**An illustration of mean waiting times for participants grouped according to the WT strategy**.

The second group of patients was found to scale their waiting times to the duration of the interval (see Figure 6). Waiting time was significantly longer for interval duration 2.5 s compared to 1.5 s [repeated measures ANOVA, *F*(1,2) = 56.99, Pillai’s Trace = 0.96, *p* = 0.02, η^2^ = 0.96]. The time of the inter-beat interval was filled by both waiting in the target zone and then moving toward the other target on the opposite side. Again, no effect of ID or movement amplitude was observed on the mean waiting times, *p* > 0.05.

There was no difference between the means of the waiting times for the different conditions between the patients and the healthy control group [1.5-s interval PD group *M* = 0.94 s, SD = 0.18 s, healthy controls – *M* = 0.75, SD = 0.06; 2.5 s interval *M* = 1.91 s, SD = 0.18, healthy controls respectively *M* = 1.76, SD = 0.02, Between Subjects Effects – group Repeated Measures ANOVA *p* > 0.05]. Again, the lack of any differences between patients and healthy controls suggest that their ability to use waiting time or MT strategies to fill time between the inter-beat intervals is preserved and cannot explain their difficulties with synchronization to the beat.

### Movement Time and Velocity

**Figure 7:**
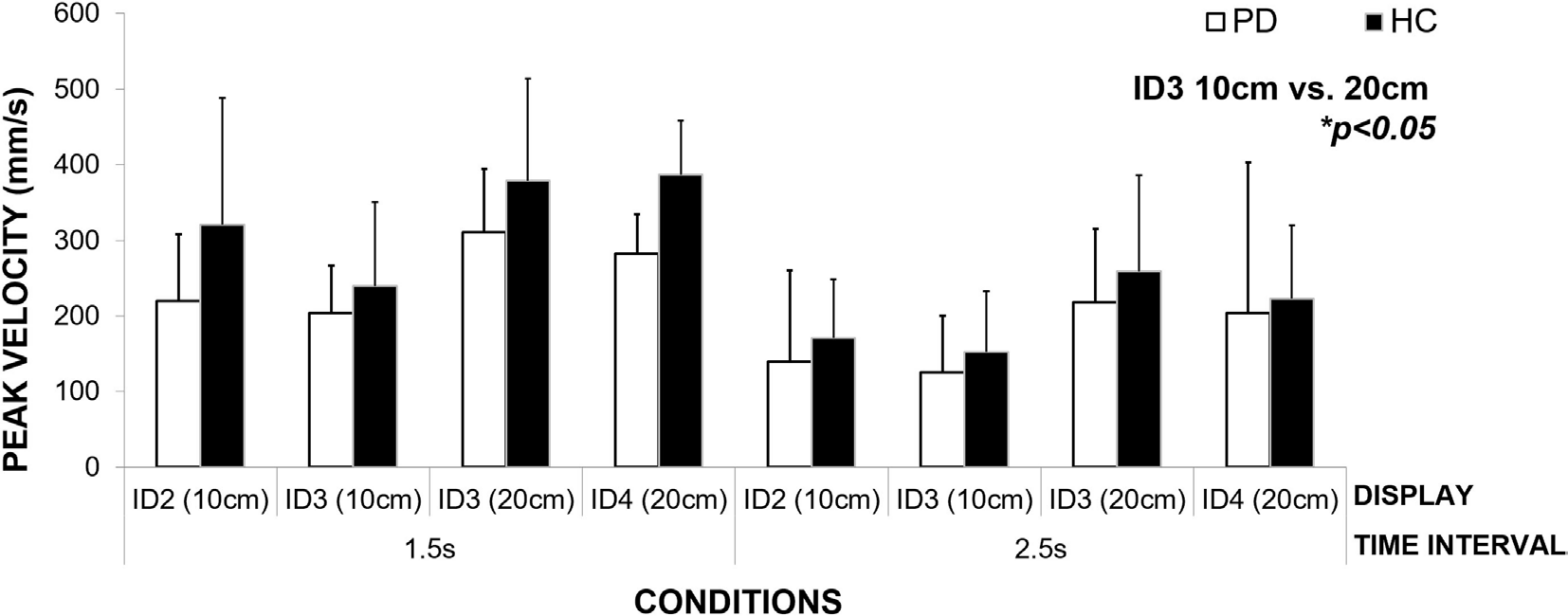
**An illustration of how the patient group adjusts the speed of their movement to meet the spatial and informational constraints of the task (size of the targets and the distance between them) in order to maintain constant movement time for each condition**.

In the previous section, we demonstrated that patients (strategy MT) maintained constant MT across a given interval duration. Our results show that to achieve this across different spatial and informational constraints of the task, they varied the speed of their movement across conditions (Figure 7). We observed a significant effect of interval duration on the peak velocity [repeated measures ANOVA *F*(1,2) = 22.74, *p* = 0.04, η^2^ = 0.92] and spatial and informational constraints of the movement [*F*(3,6) = 16.26, *p* = 0.003, η^2^ = 0.89]. Bonferroni pairwise comparisons revealed the most pronounced differences (*p* = 0.05) between the speed of the movement within the same level of difficulty – ID3 and different amplitudes of the movement – 10 and 20 cm. In comparison to healthy controls, patients moved slower in both the 1.5-s interval (23% slower), and the 2.5-s interval (15% slower) conditions, yet these differences did not reach statistical significance [*p* > 0.05 for Between Subjects Effects Repeated Measures ANOVA group vs. mean peak velocity]. Patients adjusted the speed of their movement to meet the demands of the task and despite moving more slowly than healthy adults; those differences did not reach statistical significance.

### Central Timekeeping Variance

In our study in healthy controls (26), we found an increase in central timekeeping variance (calculated with Vorberg and Wing (35) model for synchronous finger tapping) with longer interval durations (1.5 vs. 2.5 s and 3.5 s), only for those participants who used their MT as a timekeeping tool. Healthy controls who kept their MT constant and adjusted waiting time in the target zones (strategy WT), did not show an increase in central timekeeping variance.

**Figure 8:**
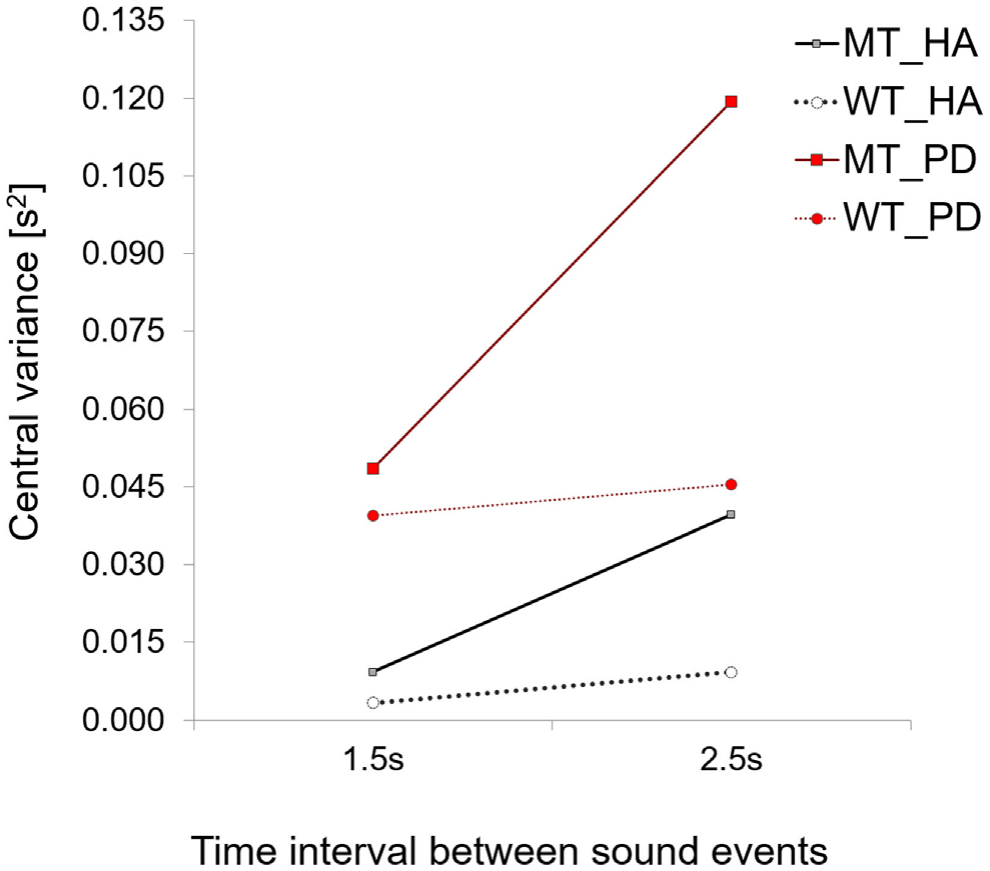
**A comparison of central variance values for PD patients and healthy adults**. Note how for both strategies central variance values are higher for PD patients when compared to healthy controls.

In this study, we found an identical increase in central timekeeping variation with the longer interval duration for PD patients who also used a MT strategy. However, the most interesting finding was that timekeeping variance was five times higher for the 1.5-s interval and three times higher for the 2.5-s interval in the patient group compared to that found for healthy controls (see Figure 8).

The effect of the group on central timekeeping variance was found to be significant [Repeated Measures ANOVA for Between Subjects Effects – across two time intervals conditions *F*(1,8) = 11.4, *p* = 0.01, η^2^ = 0.58, patients (time interval 1.5 s, *M* = 0.048, SD = 0.03, 2.5 s, *M* = 0.119, SD = 0.09) vs. healthy controls (time interval 1.5 s, *M* = 0.009, SD = 0.01, 2.5 s, *M* = 0.039, SD = 0.03)].

The difference between central timekeeping variance for the two interval durations in the patient group did not reach statistical significance, *p* > 0.05. A comparison of patients using the waiting time (WT) strategy revealed that patients had higher values of central timekeeping variance than healthy adults, but the difference was not significant [repeated measures ANOVA for between subjects effects, *p* > 0.05]. A comparison of motor variance between two interval durations did not show significant differences (1.5 s *M* = 0.05, SD = 0.06, 2.5 s *M* = 0.03, SD = 0.03) [repeated measures ANOVA *p* > 0.05]. The Lag 1 values were in the limits of (−0.5 to 0) as posited by the original Wing–Kristofferson model (duration 1.5 s, *M* = −0.03, SD = 0.03, 2.5 s *M* = −0.07, SD = 0.12). The average correction parameter α was equal to 1.17, representing the proportion of the asynchrony corrected on the adjacent motor responses (*t*-tests did not reveal any differences across two interval durations with the healthy controls sample, *p* > 0.05). Therefore an increase in variability can only be attributed to central timekeeping processes excluding factors involving the execution of the movement.

## Discussion of the Experimental Findings

### Main Findings

Our primary aim was to explore how PD patients deal with a synchronization task. Using measures of temporal accuracy, we found that with the severity of the disease, the ability to accurately synchronize movement to a sound event decreased. PD patients made greater synchronization errors when compared to healthy adult controls and their synchronization performance was more variable as measured by the spread of errors (26). In addition, patients demonstrated a tendency to underestimate the duration of the interval, rather than overestimate it, as was the case with a group of healthy adult controls. Underestimating the duration means that they tended to arrive in the target zone before the occurrence of the beat. Those results are in line with reports from other finger-tapping studies, which also showed that patients underestimate the interval duration and present a higher magnitude of temporal errors (20, 22, 36).

Difficulty with synchronization was more pronounced for the longer interval duration (2.5 s). The patient with the most advanced UPDRS score (89) was not able to coordinate movement with respect to the sound events, and as a result, the testing session was stopped after only two experimental conditions. This patient reported that it was too difficult to get into the “swing” of the movement, thus implying an inability to produce a rhythmical movement.

The second aim of our study was to explore temporal aspects of movement organization and compare findings to previous research. We wanted to investigate whether patients are able to adjust their movement to different informational, spatial and temporal demands of the task. We expected patients might organize their movement in a different way to healthy adults. Instead, all patients showed the same movement strategies as healthy adults and adjusted MT to the duration of the interval or waited in the target zone for a period of time that matched the inter-beat interval duration. In this respect, MT or waiting time was used as a tool for timekeeping. We expected that PD patients will have prolonged movement and waiting time as demonstrated by Onla-or and Winstein (16), but the difference with healthy adults was statistically not significant. We also did not find evidence that PD patients have difficulty with adjusting their movement velocity to the amplitude of the movement or target size. Patients modulated their movement velocity to meet the spatial and informational constraints of the task in the same way as healthy controls did. In line with previous research (9), we also observed that patients moved with lower velocities when compared to healthy adults, although those differences did not reach statistical significance. Neither informational load of the task, nor the amplitude of the movement had an impact on the successful performance of the task. We expected that patients might show increased difficulty when moving toward a display with a high informational load (ID = 4, two 2.5 cm × 4 cm (W × H) targets placed in 20 cm distance), but this was not the case. The informational load of the task, in line with Fitts’ speed-accuracy trade off, had an impact on the velocity of the movement between the target zones. Lower IDs triggered faster movement between target zones while higher IDs (smaller targets) forced participants to move slower between targets even though they were the same distance apart. We imposed a movement range on patients by using pre-designated target zones for intercepting the sound event. Patients did not show differences in the movement amplitude with regard to different interval durations nor did their performance differ from that found for healthy adults.

The third aim of this study was to model the difficulties with the timing of the movement. When temporal variance of the movement was split into the two components of the Vorberg and Wing (35) model of synchronization timekeeping, we found that patients had significantly higher central variance when compared to controls (five times higher for the interval duration of 1.5 s and three times higher for the interval duration of 2.5 s). This increased variability with longer interval durations is characterized by patients adjusting their movements to the interval duration. Increased variability of central timekeeping was previously reported in studies using the finger-tapping paradigm (1, 20, 37). Similarly to what our results show, the severity of the disease led to greater variability in interval reproduction (22, 38). We did not, however, observe an increase in motor variance with longer interval durations, confirming that increased variability can only be attributed to timekeeping mechanisms.

An unexpected finding was that patients at advanced stages of the disease tended not to synchronize their movement so that it ended with the beat, but instead, used the beat as a trigger to start the movement. Each time the experimenter observed this, patients were reminded that this was not the purpose of the task and were encouraged to synchronize their movement so that it ended with sound events. In PD, coupling a movement onto an external temporal framework (such as a metronome) is known to improve movement, such as gait, and is referred to in the literature as cueing (39–45). However, we explicitly asked patients to stop at the occurrence of the sound event, not initiate their movement. Our interpretation of this behavior is that as patients suffer from difficulties with movement initiation they used the beat as a cue to start moving. Patients claimed they switched to synchronizing the start of the movement with the beat unconsciously.

### Interpretation of Findings

Our results suggest that degeneration of basal ganglia circuitry might undermine the temporal prediction ability i.e., anticipating when something is going to happen in the near future. Those difficulties seem to be independent of the range of movement and increase with stretching the time interval before the occurrence of the event. To our knowledge, this is the first time that a study has addressed sensorimotor synchronization in PD patients in the context of a beat interception task based on aiming movement. Previous work by Diedrichsen et al. (22) has demonstrated a decreased ability of patients’ to synchronize finger-tapping movements to a metronome. Our results do not support the dissociation of timing proposed by Spencer and Ivry (2). Our task was event-based, yet patients demonstrated an increase in variability in both synchronization errors and timekeeping when compared to healthy adults. Spencer and Ivry (2) argued that the basal ganglia plays a minimal role in event-based timing, but is highly involved in continuous timing. Our results agree with Wing (46) proposal that both structures like the cerebellum and basal ganglia might play an important role in motor timing processes. Other authors also posited that the basal ganglia are involved, not only in the generation of “internal beats” as suggested by finger-tapping studies (47, 48), but also in the perception of complex rhythm (23). Activation of the basal ganglia during discrete timing tasks has also been reported in neuroimaging studies (fMRI), but in contrast to Spencer and Ivry (2) findings, this has not been found during temporal prediction tasks that involve continuous timing (49). Diedrichsen et al. (22) posited that basal ganglia structures are involved in error correction processes. We did not find significant differences between the error correction parameter in the group of patients and healthy adults using the Vorberg and Wing (35). Therefore, the role of the basal ganglia in the temporal control of movement and consequences of the neurodegeneration in PD on timing remains open to debate.

It is also important to note that we did not manipulate in any way the patients’ medication schedule. All of our patients who participated were on normal doses of their medication. Previous research shows that patients show higher variability in timing when tested 24 h after a break in their medication schedule (20). Deprivation of dopamine supplementation may compromise basal ganglia function to an even greater extent. In spite of a regular medication scheme, our study showed that patients with PD performed significantly worse in our synchronization task compared to healthy adult controls. Many other studies exploring event-based timing in PD were based on testing patients on medication (22, 47, 48, 50). Testing patients “off” medication would allow us to further explore the pattern of synchronization difficulties in PD. This however, would have to be conducted in a clinically supervised setting.

There are two major limitations to this study. First of all, we tested a relatively small sample of patients, which does not allow us to draw final conclusions about the causal relationship between the severity of the disease and ability to synchronize. We aim to replicate this study with a large sample of patients to validate reported findings here. Second, a limitation is the comparison of PD performance to the group of young, healthy controls, not to elderly matched controls. Although there is a convincing evidence that ability to synchronize is preserved and not significantly different in healthy aging (28, 51), we aim to include elderly controls in the replication of this study. We would suggest that a variation of this type of task could be employed by healthcare practitioners to monitor the severity of PD symptoms and be one of a large number more objective behavioral markers that look at disease progression.

## Conclusion

We found preliminary evidence that patients suffer from specific difficulties with event-based timing, namely synchronization with an external acoustic beat. This type of task requires prospective motor control (i.e., coupling movement to neural based dynamic information that helps anticipate when the beat is going to sound) and also efficient error correction processes that help tune the unfolding movement so it is in synchrony with the sounding of the next beat. By imposing a range of movement for participants, we have employed a different experimental paradigm to that used in other discrete timing experiments [e.g., Ref. (20, 22, 36, 52, 53)]. Although all of the participants moved more slowly than the healthy adult controls, they did tend to use similar strategies when performing the task and also showed that movement amplitude remained uncompromised. This enabled us to explore how temporal control varied within the controlled spatial parameters of the movement. Indeed, we found that a decrease in the temporal control of the movement seems to be independent of a decreased scaling of the movement as observed in bradykinesia, or information load of the task, but links to impaired ability to predict when something is going to happen.

## Author Notes

Correspondence concerning this paper should be addressed to MB, UMR 7287 CNRS & Université d’Aix-Marseille, Faculté des Sciences du Sport, CP 910, 163, av. de Luminy F-13288 Marseille Cedex 09, France.

## Conflict of Interest Statement

The authors declare that the research was conducted in the absence of any commercial or financial relationships that could be construed as a potential conflict of interest.
